# Passwords Usage and Human Memory Limitations: A Survey across Age and Educational Background

**DOI:** 10.1371/journal.pone.0051067

**Published:** 2012-12-05

**Authors:** Denise Ranghetti Pilar, Antonio Jaeger, Carlos F. A. Gomes, Lilian Milnitsky Stein

**Affiliations:** 1 Department of Psychology, Pontifical Catholic University of Rio Grande do Sul, Porto Alegre, Rio Grande do Sul, Brazil; 2 Department of Human Development, Cornell University, Ithaca, New York, United States of America; University Of São Paulo, Brazil

## Abstract

The present article reports a survey conducted to identify the practices on passwords usage, focusing particularly on memory limitations and the use of passwords across individuals with different age and education backgrounds. A total of 263 participants were interviewed, with ages ranging from 18 to 93 years, and education level ranging from grade school to graduate degree. Contrary to our expectations, effects of cognitive decline due to aging were not observed on memory performance for passwords. The results suggested instead, that the number of password uses was the most influential factor on memory performance. That is, as the number of circumstances in which individuals utilized passwords increased, the incidence of forgotten and mixed-up passwords also increased. The theoretical significance of these findings and their implications for good practices on password usage are discussed.

## Introduction

Before checking the balance in a bank account or sending an email to a friend, people are almost always required to enter a “secret” password to obtain access to these systems. These ubiquitous password requirements from computer based systems have the goal of avoiding unauthorized access to personal and often sensitive information. To be effective on this goal, however, the required passwords should be significantly difficult to be guessed by potential intruders [1]. As a consequence, secure passwords are typically composed of uppercase and lowercase letters combined with numbers and special characters, and are at least six characters long [2], [3], [4], [5]. The intricate characteristics of secure passwords, however, posit an unfortunate problem for password users. That is, whereas such passwords are difficult to be guessed by intruders, they are in general considerably difficult to be remembered by authorized users [6].

As extensively shown by prior human memory research, people tend to exhibit better memory performance for the gist meaning of a past event than for its details [7], [8], [9], [10]. Although secure passwords can be related to meaningful information (e.g., the name of a family member), retrieval of its meaning content is usually not a sufficient condition to access secured information because passwords must be entered verbatim, which requires knowledge about its source (the system in which one should use a particular password) and structure (the precise organization of letters, numbers, and symbols that composes a password). Therefore, recommendations for creating secure passwords end up requiring users to retrieve memories for detailed (verbatim) information, which by their turn, fade quickly with the passage of time [11] and are very susceptible to interference [12], [13].

Prior research has shown that to circumvent the difficulty in learning and remembering secure passwords, people acquire several inappropriate practices on generating and storing their personal passwords [14]. These practices include choosing passwords of personal significance, passwords short in length [15], excessively simple passwords, reusing passwords, and writing down passwords [16]. In a study in which 860 password users at the American Department of Defense were surveyed [17], it was found that long passwords are not necessarily harder to recall than short ones, but passwords composed of various kinds of characters are in fact more difficult to remember and more likely to be written down than passwords composed of only one kind of character. In some cases, inappropriate password usage habits persist even after users are lectured about computer and information security issues ([18], although see [19]).

Understanding the types of inappropriate practices that emerge from imposing security constraints on password creation is one among many aspects about password usage that might inform and improve password-based authentication procedures. Other aspects that have been the focus of research are the source, composition, and number of passwords. A survey in which 218 young adults were inquired about the source of their passwords (e.g., bank or e-mail accounts), the information contained in each password and to whom it might refer (e.g., the user’s own name or birth date), and the number of passwords [14], revealed that the vast majority of the participants (97%) used passwords to access their email account, followed by voice mail (86%) and ATMs (83%). Regarding password composition, roughly half of the passwords (53%) contained information about a name or a date, and most of the passwords (74%) referred to either the participants themselves or their relatives. Moreover, although respondents reported using 8.2 passwords on average, the average number of *unique* passwords was actually 4.5, which shows that, on average, each unique password is reused roughly once (e.g., people might use the same password to access an e-mail account and a voice mail).

This survey [14] was an important step in comprehending password users’ behavior. Nevertheless, by surveying a homogeneous sample with respect to age and education (all participants were undergraduate students enrolled in a Psychology course); it was not possible to make inferences about the impact of factors such as age and education on password usage. Thus, since it is well known that older adults typically show a general decline in memory performance, especially in tasks involving free and cued recall [20], [21], [22], [23], questions can be raised about whether older adults might show higher rates of passwords forgetting relative to younger adults. Similarly, even though fewer years of education has been shown to be associated with memory disadvantages, as faster age-related memory decline ([24], [25], [26], however see [27]), it is currently unknown whether years of education influences password usage performance. Assuming that there is an increasing amount of password users worldwide (since passwords are required to log in email accounts, bank accounts, credit card accounts, and so on), and most current password users do not posses college education (approximately 6.7% of the global population has a college degree, [28]), it is important to look into the typical password-use habits and password-related memory issues of individuals without such higher education in order to draw a more accurate picture regarding password usage in the general population.

Therefore, in the present study we surveyed the password usage in different age groups with different education levels in order to investigate the password usage factors previously reported [14] in a more diverse sample. We expected that differences in password usage would be evident across age and education level. More specifically, based on findings from extensive prior studies on memory and aging (e.g., [21], [29]), we expected that older adults would report a higher rate of forgotten passwords, an expectation that was surprisingly unconfirmed.

## Methods

### Participants

A sample of 263 individuals (150 female) from the city of Porto Alegre, Brazil, participated in the study (Table 1). Written informed consent was obtained in accordance with the Brazilian Committee for Ethics on Human Research and approved by the Institutional Review Board of the Pontifical Catholic University of Rio Grande do Sul. Three age groups were considered [30]: young adults (18–39 years old; mean, 28.3; *SD*, 6.8), middle-aged adults (40–64 years old; mean, 54.6; *SD*, 7.4), and older adults (65–93 years old; mean, 72.2; *SD*, 6.2). For each age group, participants were further divided according to education, namely, those who had not completed high school, those who had completed high school but had not entered college, and those who had at least some college education. Detailed distribution of participants by age and education is shown in Table 1.

**Table 1:** Number of participants by age and education.

	Education			
	< High	High	College	Total
18–39 years	29	10	52	91
40–64 years	50	18	32	100
65–93 years	35	17	20	72
Total	114	45	104	263

*Note:* < High = without high school degree, High = high school degree only, College = at least some college education.

## Materials

The survey instrument was based on the questionnaire developed by Brown and colleagues [14]. It was adapted through a preliminary survey conducted to determine the most frequently used password categories. The adapted questionnaire was adjusted after pilot testing with a sample of 20 participants. The first section of the questionnaire includes demographic information, such as sex, age, and education level. The second section of the questionnaire contained questions about passwords. For each password reported, participants stated its source (e.g., bank, e-mail, and mobile), number and type of characters, whether the password was chosen by themselves or assigned to them by the system, and the type of information it contained (e.g., birth date of a relative, or his/her own telephone number). The third section of the questionnaire included questions about whether participants ever forgot or mixed-up at least one password, how frequently they were required to replace their passwords, if ever, and whether they wrote any of their passwords down.

### Procedure

Participants were interviewed at various locations, such as their work places, community centers, or schools. The locations were enlisted by their entries in telephone number listings, and were then selected by the researchers according to their readiness to become involved in the research. Once the personnel responsible for the locations allowed the researchers to perform the interviews in their institutions, dates for the interviews were arranged. Participants, who had no previous contact with the experimenters, were randomly selected on these locations. The experimenters introduced themselves to the participants and gave a brief overview of the interview procedures. Once the person agreed to volunteer, he or she would sign the informed consent form before the experimenter proceeded to the interview. For each password use category on the questionnaire, participants were asked whether they used passwords for it. If so, for each password, the interviewers asked for more information about the password, such as password length and composition. As the participants responded to the questions, the interviewers wrote down their responses in the questionnaire form. The number of participants present in the locations where the interviews were conducted could vary from one participant in some cases, to up to around 20 participants in others (e.g., in a community center). In order to ensure that the participants’ privacy was protected, the interviewers were especially careful with the instructions regarding memorization strategies, thus offering plenty of detailed explanation and examples, so that the strategies provided by the participants were sufficiently generic and did not reveal their personal passwords. We also informed the participants that their responses would be anonymous and securely stored. The interviews took approximately 10 to 15 minutes.

## Results

In order to fulfill our goals, we performed analyses contrasting password usage characteristics as a function of age and education. Further analyses were conducted to examine the potential influence of age and education on reports of memory-related problems on the usage of passwords, such as password forgetting or mix-up.

### Password Characteristics and Uses

From a total of 1415 unique passwords, the majority (62.6%) was numeric only, followed by alphabetic only (24.3%), and alphanumeric (12.4%). Only 0.7% contained a combination of numbers, letters, and other characters. Among all passwords, 70% were generated by the users while 30% were generated by the systems. Overall, the participants had on average 5.38 (*SD = *3.79) password uses, and a mean of 3.98 (*SD* = 2.37) unique passwords.

As can be seem in Table 2, the mean number of password uses apparently differed across age and education levels. As revealed by an ANOVA with factors of age and education, however, while the mean number of password uses differed as a function of education, *F*(2,253) = 35.98, *p*<.001, η_p_^2^ = .22, it did not differ significantly as a function of age, *F*(2,253) = 1.42, *p* = .24, η_p_^2^ = .01. There was no interaction between age and education for the number of password uses, *F*(2,253) = 1.83, *MSE = *10.97, *p* = .12, η_p_^2^ = .028, and a follow-up t-test showed that participants with some college education had more password uses than participants with a high school degree only, *t*(147) = 4.20, *p*<.001, *d* = .80, and participants with high school degree only had significantly more password uses than participants without high school degree, *t*(157) = 2.43, *p*<.016, *d* = .59.

**Table 2:** Mean number of password uses and unique passwords according to age and education.

		Education			
		< High	High	College	Total
	18–39years	3.66 (2.30)	6.80 (3.36)	7.46 (3.21)	6.18 (3.41)
Pass.uses	40–64years	3.96 (3.24)	3.56 (1.58)	8.00 (3.62)	5.18 (3.68)
	65–93years	3.09 (1.69)	4.82 (2.86)	7.25 (6.57)	4.65 (4.23)
	Total	3.61 (2.62)	4.76 (2.79)	7.59 (4.13)	
	18–39years	2.72 (1.49)	5.00 (2.11)	5.08 (1.94)	4.32 (2.11)
Uniquepass.	40–64years	3.12 (2.02)	2.94 (1.35)	6.09 (3.18)	4.04 (2.74)
	65–93years	2.60 (1.24)	3.65 (2.00)	4.85 (2.54)	3.47 (2.07)
	Total	2.82 (1.68)	3.67 (1.92)	5.32 (2.52)	

*Note:* Standard deviations are in parentheses. < High = without high school degree, High = high school degree only, College = at least some college education, Pass. uses = password uses, Unique pass. = unique passwords.

Turning to the number of unique passwords (see Table 2), an ANOVA with factors of age and education revealed a main effect of education, *F*(2,253) = 36.98, *p*<.001, η_p_^2^ = .22. Although no effect of age was found, *F*(2,253) = 1.22, *p* = .30, η_p_^2^ = .01, a significant interaction between age and education was yielded, *F*(4,251) = 2.88, *MSE = *4.2, *p*<.023, η_p_^2^ = .04, which reflects a noticeable increase in the number of unique passwords for participants with only a high school degree who were younger (18–39 years of age) relative to the remainder participants with that education level (see Table 2). Follow-up t-tests taking only education into account showed that the mean number of unique passwords was significantly higher for participants with at least some college education than for participants with a high school degree only, *t*(147) = 3.40, *p*<.001, *d* = .75, and for participants with high school only than to participants without a high school degree, *t*(157) = 2.62, *p*<.01, *d* = .45.

Overall, these findings suggest that individuals with higher education possess noticeably higher amounts of password uses and unique passwords than individuals with less education. Even though this finding will be further examined in the following sections, the question arises of whether differences in length and uses are also present when age and education groups are compared.

Thus, in order to address the question of whether differences in password length were evident across age and education groups, we initially summed the number of characters of all passwords owned by each individual and divided it by the total number of passwords he or she had, thus yielding the average password length for each person. For the full sample, the mean password length was 4.89 characters (*SD = *1.06). Interestingly, the younger tended to have longer passwords than the older individuals [young adults = 5.24 (*SD* = .91), middle-aged adults = 4.78 (*SD* = 1.09), older adults = 4.61 (*SD* = 1.10)], a pattern that was corroborated by a One-way ANOVA, *F*(2,260) = 8.52, *MSE = *1.1, *p*<.001, η_p_^2^ = .06. Follow-up *t* tests, however, indicated that this pattern was only present when young adults and middle-aged adults were contrasted, *t*(189) = 3.17, *p*<.002, *d* = .46, [middle-aged adults vs. older adults, *t*(170) = 1.01, *p* = .315, *d* = .15].

As revealed by a One-way ANOVA, longer passwords were also adopted by individuals with higher levels of education, *F*(2,260) = 17.70, *MSE* = 1.0, *p*<.001, η_p_^2^ = .12. As showed by follow-up t-tests, even though participants with college education (mean password length = 5.33, *SD* = .93) possessed significantly longer passwords relative to participants with high school only (mean password length = 4.82, *SD* = 1.08), *t*(147) = 2.94, *p*<.004, *d* = .51, and participants with high school only possessed numerically longer passwords than participants without a high school degree (mean password length = 4.52, *SD* = 1.04), the difference between the latter groups did not reach significance, *t*(157) = 1.59, *p* = .11, *d = *.28. Overall, the findings regarding password length suggest that younger and better educated individuals have considerably longer passwords than older and less educated participants.

Regarding the type of password uses, most participants reported having at least one password for their bank account (98.1%), followed by 38.0% reporting having passwords for their credit/debit cards, 36.1% reporting using passwords to access their email accounts, and 22.8% using passwords for internet access. The data for the type of password uses for each age and education group are shown on Table 3. These data seem somewhat different than the data from prior reports [14], an issue that will be further approached in the Discussion section.

**Table 3:** Percentage of participants using each type of password according to age and education.

	< High			High			College		
	18–39 y	40–64 y	65–93 y	18–39 y	40–64 y	65–93 y	18–39 y	40–64 y	65–93 y
Bank	93.1	100	97.1	100	100	100	96.1	100	100
Cards	44.8	32.0	40.0	30.0	33.3	41.2	38.5	43.7	35.0
Email	6.9	10.0	17.1	40.0	11.1	17.6	96.1	62.5	15.0
Internet	3.4	8.0	5.7	40.0	0.0	11.8	67.3	31.2	10.0

*Note:* < High = without high school degree, High = high school degree only, College = at least some college education, 18–39 y = 18 to 39 years old, 40–64 y = 40 to 64 years old, 65–93 y = 65 to 93 years old.

### Memory Difficulties in using Passwords

In order to calculate the percentage of participants who had already experienced memory difficulties in using passwords, we considered the sum of the responses “already forgot passwords”, “mixed passwords up”, and “both forgot and mixed up passwords” [14].

Seventy two percent of the present sample (189 participants) reported experiencing memory difficulties in correctly remembering password uses. As can be seen in Table 4, it seems that reports of memory difficulties are most frequently found among people younger than 64 years of age for the group with high school degree only, as well as for all age groups for individuals with college education. Individuals without high school degree, on the other hand, reported a smaller amount of password-related memory difficulties regardless of their age. Interestingly, as can be observed by contrasting tables 2 and 4, the groups reporting more memory difficulties seem to be also the groups reporting having more password uses and unique passwords, with perhaps the exception of participants with high school degree only with ages ranging between 40 and 64. To quantitatively examine this observation, we conducted point-biserial correlations with factors of reports of memory difficulties (yes/no), number of password uses and number of unique passwords. Positive correlations were yielded between both memory difficulties and number of password uses, *r* = .26, *p*<.001, and memory difficulties and number of unique passwords, *r* = .29, *p*<.001, giving support for the observation that the frequency of memory difficulties reports is enhanced as the number of password uses and unique passwords increases.

**Table 4:** Percentage of participants reporting memory difficulties according to age and education.

	Education			
	< High	High	College	Total
18–39 years	65.5	100.0	80.8	78.0
40–64 years	64.0	83.3	87.5	75.0
65–93 years	57.1	41.2	80.0	59.7
Total	62.3	71.1	82.7	71.9

*Note:* < High = without high school degree, High = high school degree only, College = at least some college education.

To further examine the association between number of passwords and memory difficulties, we have broken down the percentage of participants reporting memory difficulties according to the amount of password uses for the three education groups. As can be seen in Table 5, there is a substantial increase in the percentage of participants reporting memory difficulties as the number of passwords reported by them increases from up to 3 passwords to between 4 and 6 passwords. Strikingly, while participants without college education who reported having between 7 and 9 passwords reported less frequently having memory difficulties than participants having between 4 and 6 passwords in those groups, participants with college education who had between 7 and 9 passwords showed the inversed pattern, that is, an increased amount of memory difficulties relative to participants having between 4 and 6 passwords. This pattern suggests that while there is a general increase in memory difficulties when the number of password uses is increased from up to 3 to between 4 and 6, and a decrease in the report of memory difficulties when the number of passwords is further increased to between 7 and 9, this trend does not hold for individuals with college education, who showed a consistent increase in the reports of memory difficulties as the number of password uses increases.

**Table 5:** Percentage of participants reporting memory difficulties according to number of password uses and education.

		Education			
		< High	High	College	Total
	1–3	49.2	63.2	58.3	53.1
Pass. uses	4–6	82.1	80.0	79.4	80.7
	7–9	75.0	62.5	91.2	84.0

*Note:* The percentages on each table bin are based on the data of at least 8 participants. < High = without high school degree, High = high school degree only, College = at least some college education, Pass. uses = number of password uses.

Interestingly, perhaps to compensate for the high frequency of memory difficulties reports in all age and education groups, a substantial amount of participants from all groups reported keeping a physical record or having to reset passwords at least once (see Table 6). Therefore, while at least a physical record of the passwords were kept by 59.8% of the participants reporting memory difficulties, a physical record was kept by only 44.6% of the participants not reporting memory difficulties. Similarly, 67.7% of the participants reporting memory difficulties had to reset passwords, at least once, while only 21.6% of the participants not reporting memory difficulties had to reset passwords.

**Table 6:** Percentage of participants reporting keeping physical records of passwords and having to reset passwords according to age and education.

		Education			
		< High	High	College	Total
Physicalrec.	18–39 years	48.3	40.0	32.7	38.5
	40–64 years	68.0	66.7	65.6	67.0
	65–93 years	60.0	35.3	75.0	58.3
	Total	60.5	48.9	51.0	
Resetpass.	18–39 years	44.8	90.0	55.8	56.0
	40–64 years	50.0	50.0	71.9	57.0
	65–93 years	40.0	41.2	75.0	50.0
	Total	45.6	55.6	64.4	

*Note:* < High = without high school degree, High = high school degree only, College = at least some college education, Physical rec. = Participants who kept a physical record of at least one password, Reset pass. = Participants who reported having to reset at least one password.

Overall, the present findings suggest that the amount of password uses as well as the amount of unique passwords one possesses has an essential role in the incidence of forgetting and mixing up passwords. Contrary to our expectations, however, age and education level did not affect reports of memory difficulties as much as number of passwords did. Having numerous passwords, therefore, seems to be the main reason leading people to experience memory difficulties regarding their passwords.

## Discussion

The present survey showed that although the mean number of password uses and unique passwords did not change significantly as a function of age, it did change as a function of education, suggesting that more educated individuals possess higher amounts of password uses and unique passwords. Regarding password length, however, both age and education led to significant changes. That is, younger individuals as well as more educated individuals reported having more passwords than older and less educated individuals. Overall, the participants reported using passwords mainly for activities related to bank, cards and internet access (including email). Notably, younger and more educated individuals reported having more passwords to internet related activities than the other groups, as can be seen in Table 3. In terms of reports of memory difficulties, the amount of passwords one possesses, and not age, seems to be the main predictor of whether or not password forgetting or password mixing up will arise. This latter finding is surprising, since we predicted that age would be a critical factor on the frequency of forgetting and mixing up passwords.

Overall, our data regarding the number of passwords used by the participants differ from the data reported by Brown and colleagues [14]. Critically, whereas the mean number of password uses and unique passwords in the present experiment are 5.38 and 3.98 respectively, the mean number of password uses and unique passwords reported by those authors are 8.18 and 4.45 respectively. A potential reason for this discrepancy is that those authors interviewed college students only, whereas here participants with different age ranges and education levels were recruited. Thus, if we look into the number of password uses and unique passwords from the group with ages between 18 and 39 and with at least some college education, our data become much more similar to the data reported by those authors (mean number of password uses = 7.46, mean number of unique passwords = 5.08). Furthermore, when we analyze separately the participants who were currently college students at the time of the interview (21 participants), the mean number of password uses also remained similar to the mean number of password uses previously reported [14] (mean = 6.81), as well as the mean number of unique passwords (mean = 4.52), though slightly less so in contrast to when the whole group with at least some college education were contrasted with the data reported by Brown and colleagues [14]. Thus, although differences in the number of password uses and unique passwords are evident when the current data is contrasted to the data reported by those authors, this difference seems to be mostly due to age and education differences, since the data from both studies become much closer when the sample characteristics are matched.

Further differences between the present data and the data reported by Brown and colleagues [14] arise when the percentage of reports for each type of password uses are contrasted. The most salient, perhaps, being the low number of participants reporting using passwords to access their email account in the present data (36.1%) in contrast to the data reported by those authors (97%). As can be seen in table 3, however, this difference seems to be due only to the demographic discrepancies between studies. That is, as can be seen in that table, individuals with at least some college reported having roughly as many passwords to email access as the participants in the study reported by Brown and colleagues [14]. Furthermore, when individuals currently enrolled at college at the time of the interview were separately analyzed, the percentage of participants using passwords to access email accounts were actually higher than the percentage reported by Brown and colleagues [14] (100% in the present study and 97% in Brown and colleagues study).

Interestingly, as shown by the analysis of password length, younger and more educated individuals reported having significantly longer passwords than older and less educated individuals. A potential account for this difference may be related to working memory discrepancies among the age groups and the education groups. That is, the length of the passwords chosen by each given participant may have been in accordance with each participant’s own working memory capacities. Thus, as working memory capacities decline with age [31], [21], older individuals may have opted for adopting shorter passwords than younger participants. Similarly, as working memory capacities are enhanced by education and skills learning [32], less educated individuals may have opted for adopting shorter passwords than more educated individuals. Although we can only hypothesize over this issue in the present study, further research in which measurements of working memory are acquired may shed light on this topic. Specifically, further research can examine this possibility by investigating whether or not the choice for longer or shorter passwords is influenced by individual differences in working memory capacities.

Regarding the frequency with which participants reported memory difficulties, a large number of participants reported forgetting or mixing up passwords (72%) in the present sample. In the survey reported by Brown and colleagues [14], however, only 31.1% of the participants reported forgetting passwords and 22.5% reported mixing up passwords. Interestingly, the present data do not match the data reported by those authors [14] even when individuals with college education are considered, as can be seen in Table 4. The reason for such a between-studies discrepancy in the reports of memory difficulties is not yet clear, and perhaps further research could be useful to clarify such puzzling difference.

Contrary to the natural expectation of poorer memory performance for older adults, age does not seem to play a significant role in the specific case of remembering passwords. Specifically, older adults did not report more memory difficulties than their younger counterparts. A potential account for the lack of differences according to age is that older participants reported less memory difficulties than younger adults due to a lack of recollection of past memory failures. Such lack of recollection for existing past memory failures might have resulted in significantly diminished reports of memory difficulties, equating the older and the younger individuals in their reports of past memory problems. Further research utilizing experimental manipulations and various age groups might be needed to point out whether or not this account for these null effects is correct.

Our data also indicate that an impacting factor on password forgetting is the number of unique passwords owned by the users. Prior research had already pointed out that having to remember multiple passwords decreased memorability and increased the cognitive overload associated with the password processing [33]. They recommended having no more than five different passwords. Besides, by owning more passwords, people with better educational backgrounds appear to be at a higher risk of experiencing memory difficulties when using passwords, such as password forgetting and confusion. Also, prior research found that users with eight or more passwords were at a higher risk of forgetting a password at least once a month [16].

Even though recommended passwords consist typically of a mixture of numbers, letters and other characters, our findings indicated that less then 1% of the reported passwords had these characteristics. Although the participants in this study were bound by the limitations of the systems for which they used passwords, this result corroborates prior reports in which the recommendation to use all kinds of characters in a password is not usually followed [16], [17]. Interestingly, the only 10 passwords (out of 1415) containing special characters were owned by respondents with better educational backgrounds, and six of them were owned by people who were 65 to 93 years-old. Unlike other studies [16], [17], the vast majority of passwords was numeric only, followed by alphabetic only, and alphanumeric. A reason for the discrepancy between the present findings and prior data might be the password requirements typically made by Brazilian banks, which usually consists of numeric only passwords.

As suggested by the present findings, it seems that a reasonable recommendation for information security industries would be to protect information with a low number of passwords, preferentially a number that users can effectively memorize. Human factor guidelines should be available to assist people in the development of strong passwords which are both memorable and acceptable from an information security standpoint. This might be achieved by training users and raising their awareness about the risks associated with the use of weak passwords, as well as recommending good practices, such as specific mnemonic techniques [19]. Prior research [34], demonstrated that passwords generated by means of a mnemonic technique, as the first letters of a song verse (e.g., for the verse “Welcome to the Hotel California!”, create password “W2tHCa!”), can be as memorable as simple words and as secure as random strings (see also [35]). Also, if it is the number of passwords owned by the users the main source of difficulties in passwords use, it seems that the only reasonable solution is to have a number of passwords that normal adults can handle, which is a maximum of four to five passwords [15]. Since one may not be able to avoid having several passwords, Brown and colleagues’ suggestion of categorizing information seems to be a potential solution [14]. One could devise four or five categories of information, according to their importance (i.e., the inherent value of the data to the user) and sensitivity (i.e., the degree to which problems would arise if the information protected was known to others), and create four or five passwords that he/she can remember, with a strength level appropriate for each category, and then yes, reuse the passwords.

In sum, future research could test the efficacy of approaches like the ones mentioned in the previous paragraph, considering both security and memory performance. Moreover, two issues that were not examined in this study, namely, the frequency of use of a password or how long a password has been used, are likely to have an impact on memory, and their role in memory for passwords could be investigated as well. In addition, the vast body of knowledge from cognitive science in general and memory research in particular, could be used by IT system developers to make password learning and retrieval easier, avoiding the acquisition of inappropriate practices that can put at risk the systems’ security.
